# Optimizing root spatial distribution during the flower-boll stage can reduce yield losses from square stage drought in cotton

**DOI:** 10.1186/s12870-025-07346-4

**Published:** 2025-12-15

**Authors:** Congcong Guo, Xiaoyuan Bao, Hongchun Sun, Hongjuan Zhao, Lingxiao Zhu, Yongjiang Zhang, Ke Zhang, Anchang Li, Cai Zhao, Liantao Liu, Cundong Li

**Affiliations:** 1 State Key Laboratory of Aridland Crop Science, College of Agronomy, Gansu Agricultural University, Lanzhou, 730070 China; 2 State Key Laboratory of North China Crop Improvement and Regulation/Key Laboratory of Crop Growth Regulation of Hebei Province, College of Agronomy, Hebei Agricultural University, Baoding, 071001 Hebei China

**Keywords:** Root spatial distribution, Drought tolerance, Cotton, *F*_v_/*F*_m_, Yield

## Abstract

**Background:**

Root spatial distribution, an important root function phenotype, is closely related to drought tolerance. Drought is known to hinder cotton development, but, the effects of drought at the square stage on the spatial distribution characteristics of roots at the flower-boll and boll opening stages remain unclear.

**Methods:**

To address this gap, 30 different cotton cultivars were grown in the field. The control treatment received routine irrigation (1175 m^3^·hm^−2^), and the drought treatment received reduced irrigation (822.5 m^3^·hm^−2^). Multiple parameters, including root traits, above-ground traits, and yield, were assessed.

**Results:**

At the flower-boll stage, drought conditions during the square stage significantly reduced SPAD and optimal quantum efficiency (*F*_v_/*F*_m_) values in cotton. Cluster analysis categorized cultivars as drought-tolerant, relatively drought-tolerant, intermediate-sensitive, relatively drought-sensitive, or drought-sensitive cultivars. Under drought conditions, drought-tolerant cultivars exhibited a greater decrease in average lateral root angles, a less pronounced increase in root-shoot ratio, and 24.25% higher yield than drought-sensitive cultivars. Additionally, in the 50–60 cm soil layer, root length density in drought-tolerant cultivars increased by 141.78%, compared to drought-sensitive cultivars under drought conditions, and the drought-sensitive and tolerance cultivars were symmetrically distributed in the 0–30 cm soil layer. Notably, owing to substantial rainfall during the study, there were no significant differences in root spatial distribution at the boll opening stage.

**Conclusion:**

Drought at the square stage can improve drought tolerance and reduce yield losses by reducing lateral root angles, optimizing the root-shoot ratio, and increasing root length density in deeper soil during the flower-boll stage.

**Supplementary Information:**

The online version contains supplementary material available at 10.1186/s12870-025-07346-4.

## Background

Cotton (*Gossypium hirsutum* L.) is among the most important economic crops, contributing to approximately 35% of global fiber consumption [1, 2]. In recent years, the expansion of dry farmland area has paralleled the growing impact of global warming. Nearly 35% of the world’s land area and about 43% of arable land are in arid or semi-arid regions [3]. China, a pivotal global producer and consumer of cotton, grapples with drought constraints affecting cotton production within its borders [4]. Drought during the budding period is an important factor affecting the growth, development, and yield of cotton in the Yellow River Basin. Exploring root traits closely related to drought tolerance can help in both identifying drought tolerance mechanisms and screening and cultivating drought-tolerant cultivars. 

Roots are the principal organs for water uptake in plants. They are the first to detect and react to soil moisture levels, triggering responses in root morphology [5, 6]. Root distribution refers to the special variation in roots in the growing environment, e.g., as described using a gradient or grid, reflecting the relative distribution of root lengths in the soil profile [7, 8]. The root spatial distribution represents a critical aspect of root morphological traits, demonstrating rapid adjustments in response to changes in the soil moisture distribution. These adjustments aid in adaptation to limit the adverse effects of abiotic stress. Consequently, under drought stress, the spatial root distribution assumes a pivotal role in the efficient acquisition of water and the enhancement of water use efficiency [9]. The root spatial distribution is integral to the ability of plants to effectively utilize nutrients and water, thus enhancing their adaptation to various environmental changes. It reflects the intimate interconnection among root biomass, length, and soil nutrient uptake efficiency [7, 8]. In addition, root spatial distribution plays an important role in enhancing drought resistance of crop cultivars [5, 10]. For example, Chen et al. [11] emphasized that higher root length density and fine root proportion can led to higher cotton yield. Thus, the root spatial distribution serves as a pivotal determinant of the ability of plants to withstand drought by optimizing resource utilization. However, there are still certain limitations that merit further consideration, particularly concerning the impact of drought during the budding period on the root system architecture and root spatial distribution of diverse drought-tolerant cotton cultivars. 

To explore this issue, we examined the root spatial distribution, including the root-shoot ratio, number of lateral roots, lateral root angle, and root length density as well as the root biomass density of different soil layers. Additionally, we investigated above-ground characteristics, including plant height, stem diameter, and SPAD and optimal quantum efficiency (*F*_v_/*F*_m_) values, across different drought-tolerant cultivars. The objectives of this study evaluating the flower-boll and boll opening stages comprised the following aims: (1) to explore the root-shoot ratio, number of lateral roots, and lateral root angle among various drought-tolerant cotton cultivars and their correlation with drought tolerance in detail and (2) to distinctly reveal variation and interactions in root length density and root biomass density at different soil depths among this diverse collection of drought-tolerant cotton cultivars. This research provides novel analytical results on the variation in root spatial distribution among distinct drought-tolerant cotton cultivars under drought conditions during the growth stage. This thorough examination of the root responses of these cultivars provides the foundation for uncovering their unique adaptation mechanisms to drought stress, thereby laying valuable genetic and physiological groundwork for the future cultivation of drought-tolerant cotton cultivars.

## Results

### Drought impact on above-ground growth in various cotton cultivars

**Fig. 1:**
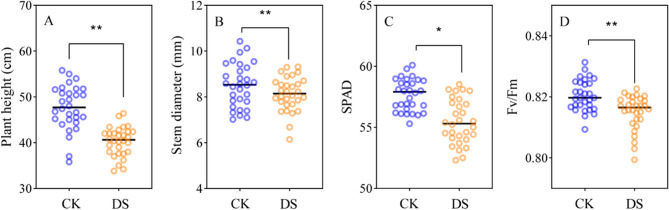
Effects of drought stress on above-ground traits in different cotton cultivars at flower-boll stage. Statistical analyses of plant height (**A**), stem diameter (**B**), SPAD (**C**) and *F*v/*F*m (**D**). CK, control; DS, drought stress. Different letters indicate statistical differences (*p *< 0.05) between treatments of different cultivars. Values are the means ± SE(n = 180)

The effects of drought stress on the phenotypic traits of above-ground agronomic characteristics were first investigated. In our assessment of 30 cotton cultivars, we observed significant inhibition of plant height (Fig. 1A), stem diameter (Fig. 1B), SPAD value (Fig. 1C), and *F*_v_/*F*_m_ (Fig. 1D) under drought stress. Specifically, compared to the control conditions, plant height, stem diameter, SPAD value, and *F*_v_/*F*_m_ value under drought stress decreased by 15.52%, 5.50%, 3.80%, and 0.67%, respectively (Fig. 1).

### Comprehensive evaluation and cluster analysis of drought tolerance of different cotton cultivars

Standardized drought tolerance coefficients were obtained for 30 cotton cultivars at the flower-boll stage (Table S1). Principal component analysis was then used to calculate the cumulative contribution of the different composite indicators by transforming the original 11 individual traits into four independent composite indicators (Table S2). The first principal component (Cl1, 29.82% contribution) was mainly associated with root spatial distribution traits, such as root length density (RLD) and root-shoot ratio. The second component (Cl2, 19.04%) reflected above-ground physiological traits, including SPAD and *F*_v_/*F*_m_ values. The third component (Cl3, 15.15%) primarily represented yield-related traits (e.g., boll weight and seed cotton yield), and the fourth component (Cl4, 9.61%) included other morphological traits.

According to Eq. (2), the membership function values of the comprehensive indicators of 30 cultivars were calculated (Table S3). In the principal component analysis, higher Cl1–4 values indicate more drought tolerance, while lower Cl1–4 values indicate less drought tolerance. According to the contribution rate of each comprehensive index, Eq. (3) was used to calculate the index weight. After calculation, the weights of the four comprehensive indicators were 0.41, 0.26, 0.20 and 0.13, respectively (Table S2). Equation (4) was used to calculate the overall drought tolerance of different cotton cultivars (Table S3), and the drought tolerance of cotton cultivars was ranked according to their *D*-value. Higher *D*-values indicate cultivars with a deeper root distribution, greater water-use efficiency, and higher yield stability under drought, whereas lower *D*-values correspond to shallower-rooted cultivars with correspondingly reduced drought resilience. (Table S4).

**Fig. 2:**
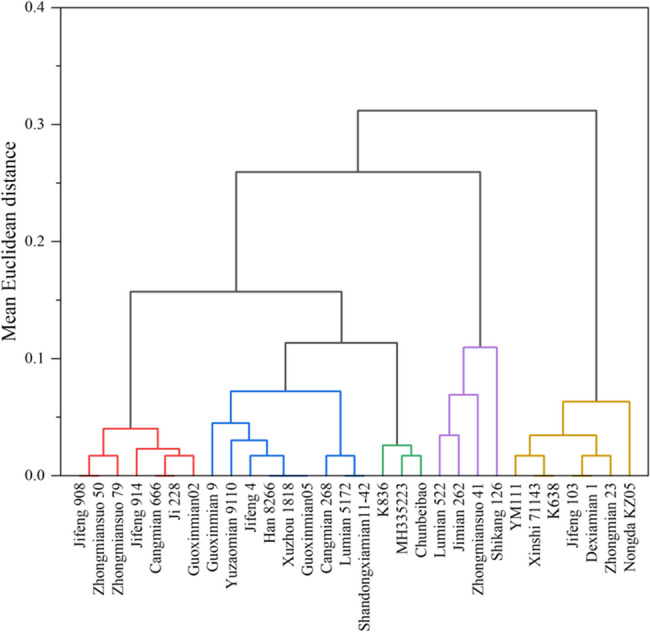
Systematic cluster analysis of 30 cotton cultivars based on D values

The *D*-values were further subjected to systematic clustering to produce a dendrogram of the 30 cultivars (Fig. 2). According to the sensitivity to drought stress, the 30 cotton cultivars can be clustered into five types at the Euclidean distance threshold of 0.1, corresponding to (1) drought-tolerant, (2) relatively drought-tolerant, (3) intermediate-sensitive, (4) relatively drought-sensitive, and (5) drought-sensitive cultivars.

### Drought impact on yield in different cotton cultivars

**Fig. 3:**
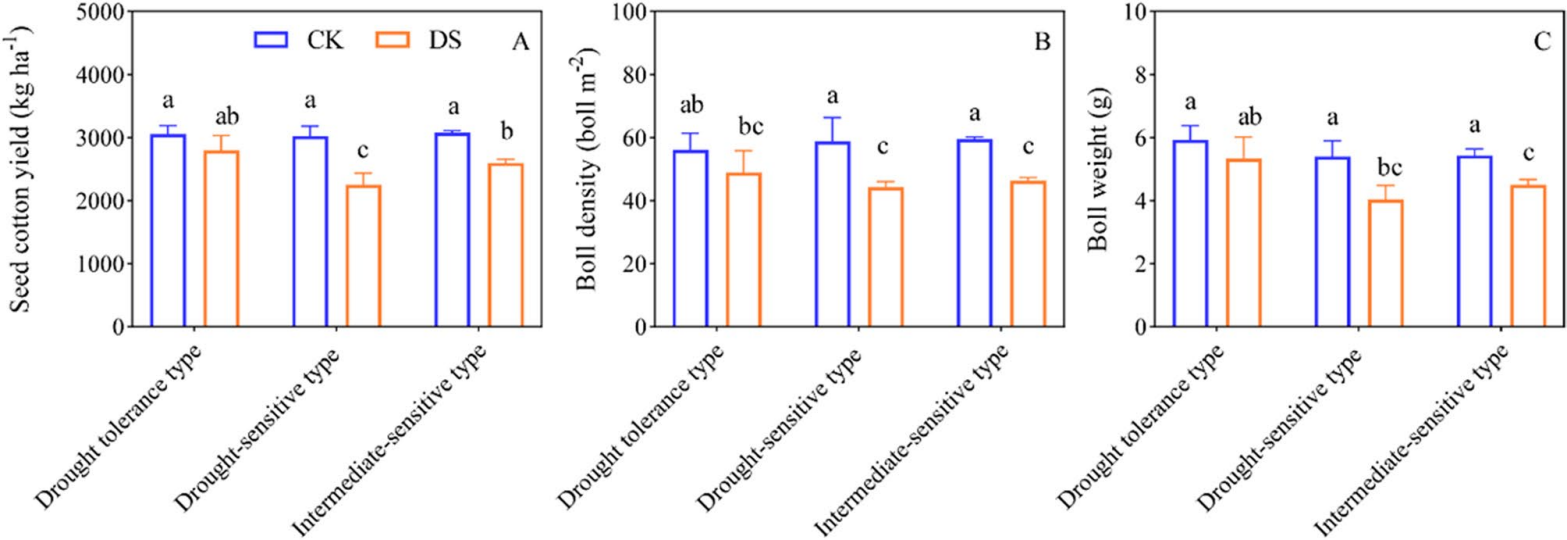
Effects of drought stress on seed cotton yield of different drought tolerant cotton cultivars. Statistical analyses of seed cotton yield (**A**), number of bolls per unit area (**B**) and boll weight (**C**). Different letters indicate statistical differences (*p* < 0.05) between treatments of different cultivars. Values are the means± SE (n = 9). CK, control; DS, drought stress

Drought stress significantly reduced cotton yield (Fig. 3). Under drought stress conditions, the seed cotton yield for drought-tolerant, drought-sensitive, and intermediate-sensitive cultivars decreased by 8.48%, 25.62%, and 15.67%, respectively, compared to the control conditions (Fig. 3A); similarly, their boll density decreased by 12.77%, 24.77%, and 21.97%, respectively (Fig.3 3B), while their boll weight decreased by 10.11%, 25.31%, and 17.18%, respectively (Fig. 3C). Additionally, variation among genotypes was observed in cotton seed yield under drought stress. Specifically, drought-tolerant and intermediate-sensitive cultivars exhibited an increase in seed cotton yield, by 24.25% and 15.47%, respectively, compared to drought-sensitive cultivars (Fig. 3A); similarly, their boll density increased by 10.63% and 4.90%, respectively (Fig. 3B), and their boll weight increased by 32.23% and 11.57%, respectively (Fig. 3C).

### Effects of drought on lateral root angle, number of lateral root, and root-shoot ratio of drought-tolerant cotton cultivars

**Fig. 4:**
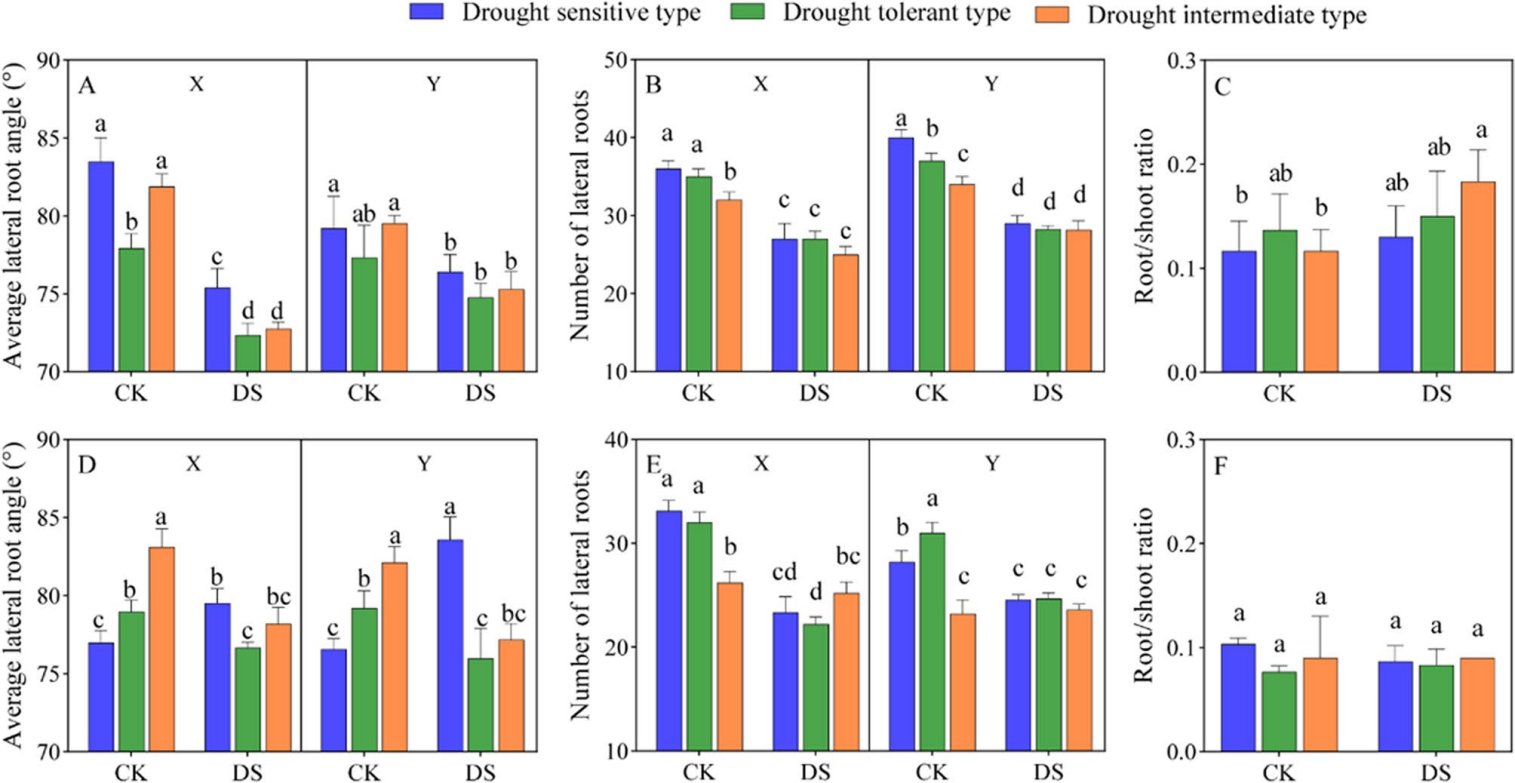
Effects of drought stress on root spatial distribution in cotton of different cultivars and orientations. Statistical analyses of average lateral root angle (**A**), number of lateral roots (**B**) and root/shoot ratio (**C**) perpendicular versus parallel to the sowing row at flower-boll stage; and average lateral root angle (**D**), number of lateral roots (**E**) and root/shoot ratio (**F**) perpendicular versus parallel to the sowing row at boll opening stage. CK, control; DS, drought stress; X, direction perpendicular to the sowing row; N, direction parallel to the sowing row. Values are the means ± SE (n = 9). Different letters indicate statistical differences (*p *< 0.05) between treatments of different cultivars

At the flower-boll stage, in samples collected parallel to the sowing rows, the average lateral root angle and number of lateral roots of intermediate-sensitive and drought-tolerant cultivars under drought stress were significantly reduced (*p* < 0.05, Fig. 4ABDE). The trend was similar between planes perpendicular and parallel to the sowing row. At the flower-boll stage, under drought stress, the average lateral root angles of the intermediate-sensitive and drought-tolerant cultivars parallel to the sowing rows were significantly (*p* < 0.05) reduced by 4.04% and 3.49%, respectively (Fig. 4A). The number of lateral roots of drought-tolerant cultivars were significantly (*p* < 0.05) reduced by 7.41%, compared to the drought-sensitive cultivars (Fig. 4B).

Drought stress promoted the root-shoot ratio of cotton. At the flower-boll stage, the root-shoot ratio of drought-sensitive cultivars increased by 36.36% under drought stress compared with their own control (Fig. 4C). There were genotypic differences in the response of root-shoot ratio to drought stress among the different drought-tolerant cotton cultivars (Fig. 4CF). At the flower-boll stage, under drought condition, the drought-tolerant and intermediate-sensitive exhibited an 18.18% higher root-shoot ratio than drought-sensitive cultivars (Fig. 4C). Since the precipitation in July was as high as about 450 mm and no drought stress occurred, there were no significant differences in lateral root angles, number of lateral roots, or root-shoot ratio among the cultivars.

### Effects of drought on root length density of different drought-tolerant cotton cultivars

**Fig. 5:**
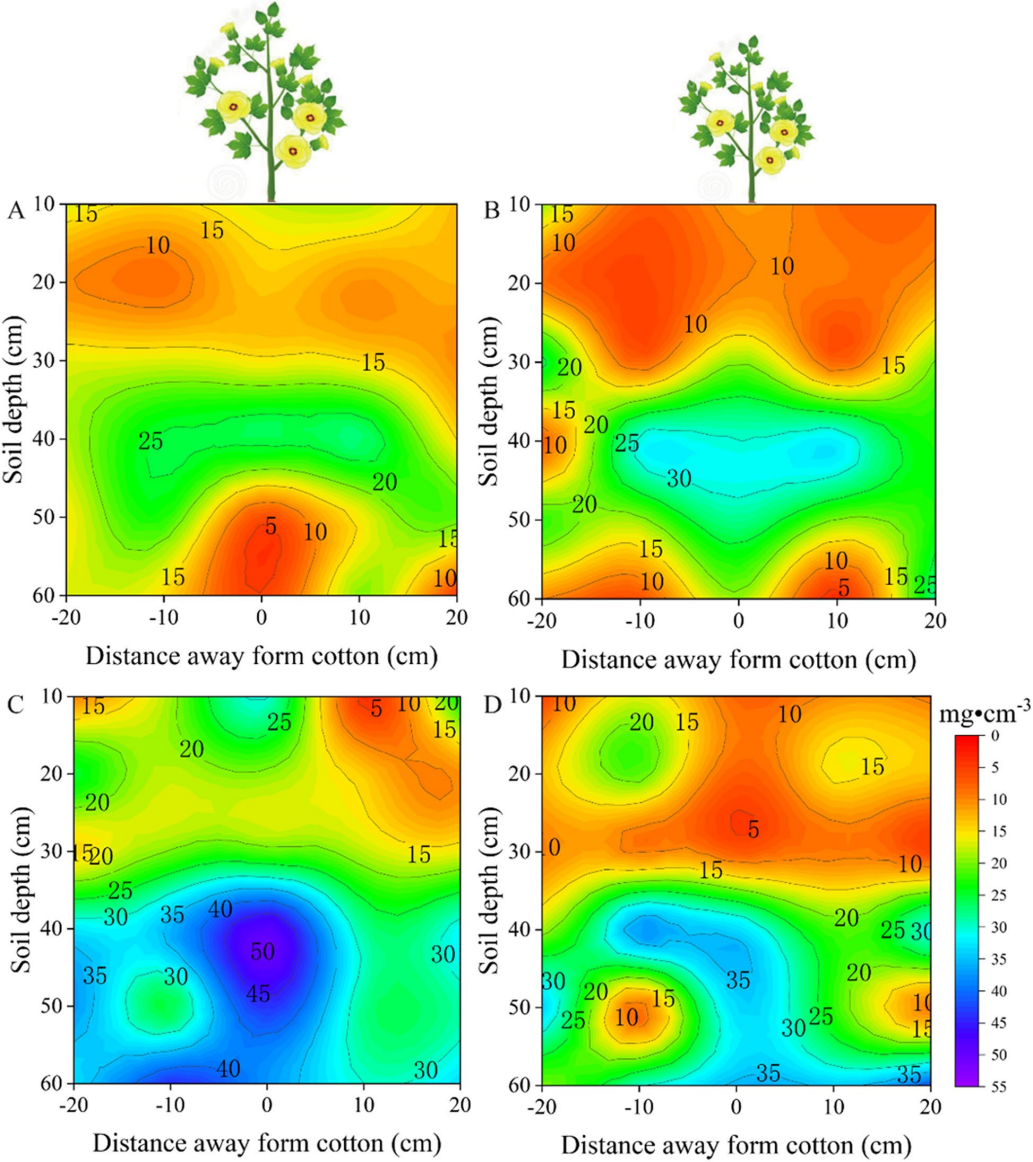
At the flower-boll stage, vertical distribution of root length density in the soil profile was affected by soil water treatment. Root length density under control-drought sensitive cultivars (**A**), drought stress-drought sensitive cultivars (**B**), control-drought tolerant cultivars (**C**) and drought stress-drought tolerant cultivars (**D**) treatments, respectively. Values are the means ± SE (n = 6)

Drought-tolerant and sensitive cultivars were selected from among 30 cotton cultivars to comparatively analyze the response characteristics of root distribution to drought stress. At the flower-boll stage, the root length density of both drought-sensitive and tolerant varieties under control conditions and drought stress increased with soil depth, Mainly up to 30cm (Fig. 5). Under control conditions, the vertical distribution of root length density of the drought-sensitive varieties was Mainly concentrated at 40–50 cm (Fig. 5A), while the vertical distribution of root length density of the drought-tolerant varieties was Mainly concentrated at 40–60 cm (Fig. 5C). Under drought stress, the vertical distribution of root length density of the drought-sensitive varieties was Mainly concentrated at 30–50 cm (Fig. 5B), while the vertical distribution of the root length density of drought-tolerant varieties was Mainly concentrated at 40–60 cm (Fig. 5D). Under drought stress, the vertical distributions of root length density of drought-sensitive and drought-tolerant cultivars were symmetrically distributed in the 0–30 cm soil layer (Fig. B5D). Under drought stress, in the 0–60 cm soil layer, the root length density of drought-sensitive varieties was 21.01% (*p* < 0.05) higher than that under the control treatment, and the degree of variation in root distribution was higher than that under the control treatment (Fig. 55B). During drought stress, the root length density in the 50–60 cm soil layer was 141.78% higher in the drought-tolerant varieties compared to the drought-sensitive varieties (Fig. 5D). This substantial increase in deep-layer root allocation is agronomically relevant, as the 50–60 cm soil layer often retains residual moisture during the late growth period, enabling drought-tolerant cultivars to Maintain photosynthetic performance and achieve a 24.25% higher yield under drought stress.

**Fig. 6:**
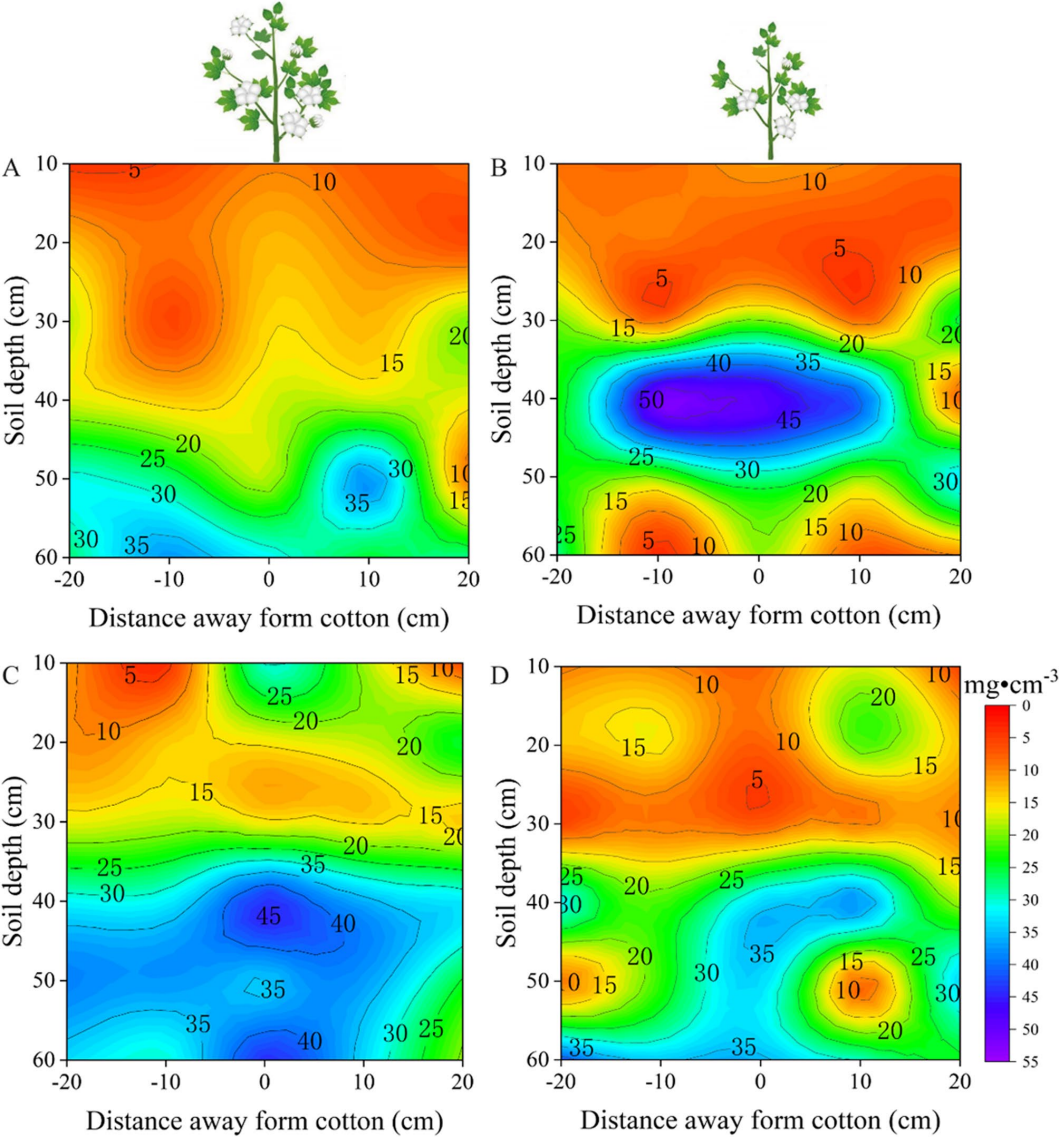
At the boll opening stage, vertical distribution of root length density in the soil profile was affected by soil water treatment. Root length density under control-drought sensitive cultivars (**A**), drought stress-drought sensitive cultivars (**B**), control-drought tolerant cultivars (**C**) and drought stress-drought tolerant cultivars (**D**) treatments, respectively. Values are the means ± SE (n = 6)

Changes in both root length density and soil depth at the boll opening stage followed the same trend as that at the flower-boll stage. Under the control conditions, the root length density of both drought-sensitive and tolerant varieties increased with soil depth (Fig. 6 AC). Meanwhile, the vertical distribution of roots in the drought-sensitive variety was Mainly concentrated at 40–60 cm (Fig. 6A), while the drought-tolerant variety exhibited a similar trend to that observed at the flower-boll stage, Mainly concentrated in the 40–60 cm layer (Fig. 6C). Under drought stress, the root length density of drought-sensitive varieties increased with soil depth, reaching a Maximum value in the 30–40 cm soil layer and then decreasing rapidly (Fig. 6B), while drought-tolerant varieties showed a gradually increasing trend (Fig. 6D). The vertical distribution of drought-sensitive variety roots was Mainly concentrated at 30–50 cm (Fig. 6B), while the vertical distribution of drought-tolerant variety roots was consistent with the trend of the root distribution at the flower-boll stage under the control treatment, i.e., Mainly concentrated at 40–60 cm (Fig. 6D). Under drought stress, the root length density of drought-tolerant varieties was wider than that of drought-sensitive varieties in terms of horizontal distribution (Fig. 6D). Furthermore, the root length density in the 50–60 cm soil layer of drought-tolerant varieties was 147.96% higher than that of drought-sensitive varieties (Fig. 6BD). Such a pronounced deep-root advantage may have allowed the drought-tolerant cultivars to exploit stable subsoil water reserves during boll filling, supporting sustained leaf function and contributing to yield stability under water-limited conditions.

### Effects of drought on root biomass density of different drought-tolerant cotton cultivars

**Fig. 7:**
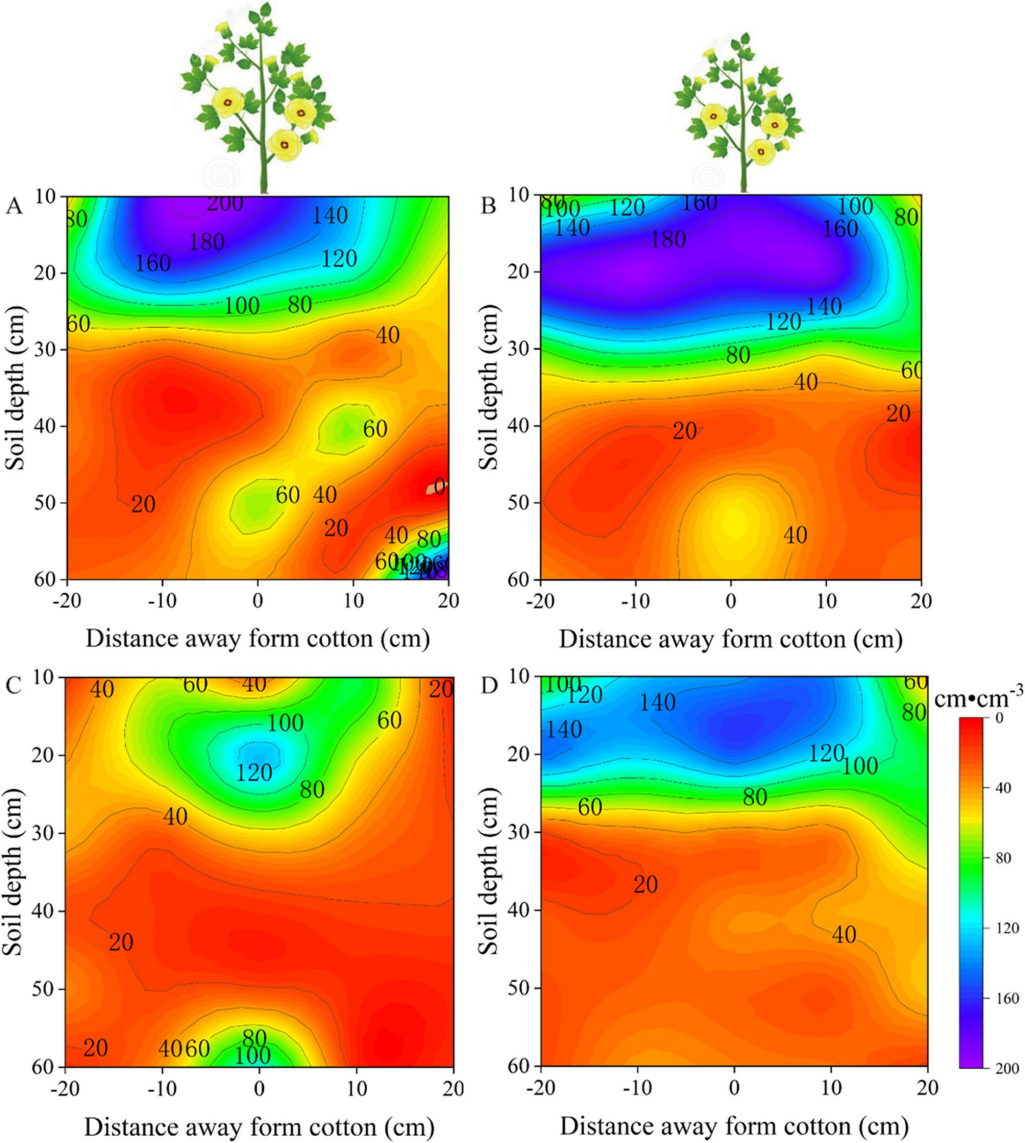
At the flower-boll stage, vertical distribution of root biomass density in the soil profile was affected by soil water treatment. Root length density under control-drought sensitive cultivars (**A**), drought stress-drought sensitive cultivars (**B**), control-drought tolerant cultivars (**C**) and drought stress-drought tolerant cultivars (**D**) treatments, respectively. Values are the means ± SE (n = 6)

At the flower-boll stage, the root biomass density distribution of drought-sensitive varieties was Mainly concentrated in the 0–20 cm layer under control conditions (Fig. 7A), while that of drought-tolerant varieties was Mainly concentrated in the 0–30 cm layer (Fig. 7C). In contrast to drought-sensitive varieties, part of the root biomass density of drought-tolerant varieties was concentrated in the deeper layer up to 60 cm deep (Fig. 7AC). Under drought stress, the distribution of root biomass density of drought-sensitive varieties was Mainly concentrated in the 0–30 cm soil layer (Fig. 7B), while the vertical distribution of root biomass density of drought-tolerant varieties was Mainly concentrated in the 0–20 cm soil layer (Fig. 7D). Root biomass density under the drought stress treatment was widely level distributed compared to that under the control; however, the biomass density of variety drought-sensitive varieties decreased by 58.47% (*p* < 0.05, Fig. 7AB), while that of drought-tolerant varieties decreased by 36.75% (*p* < 0.05, Fig. 7CD).

**Fig. 8:**
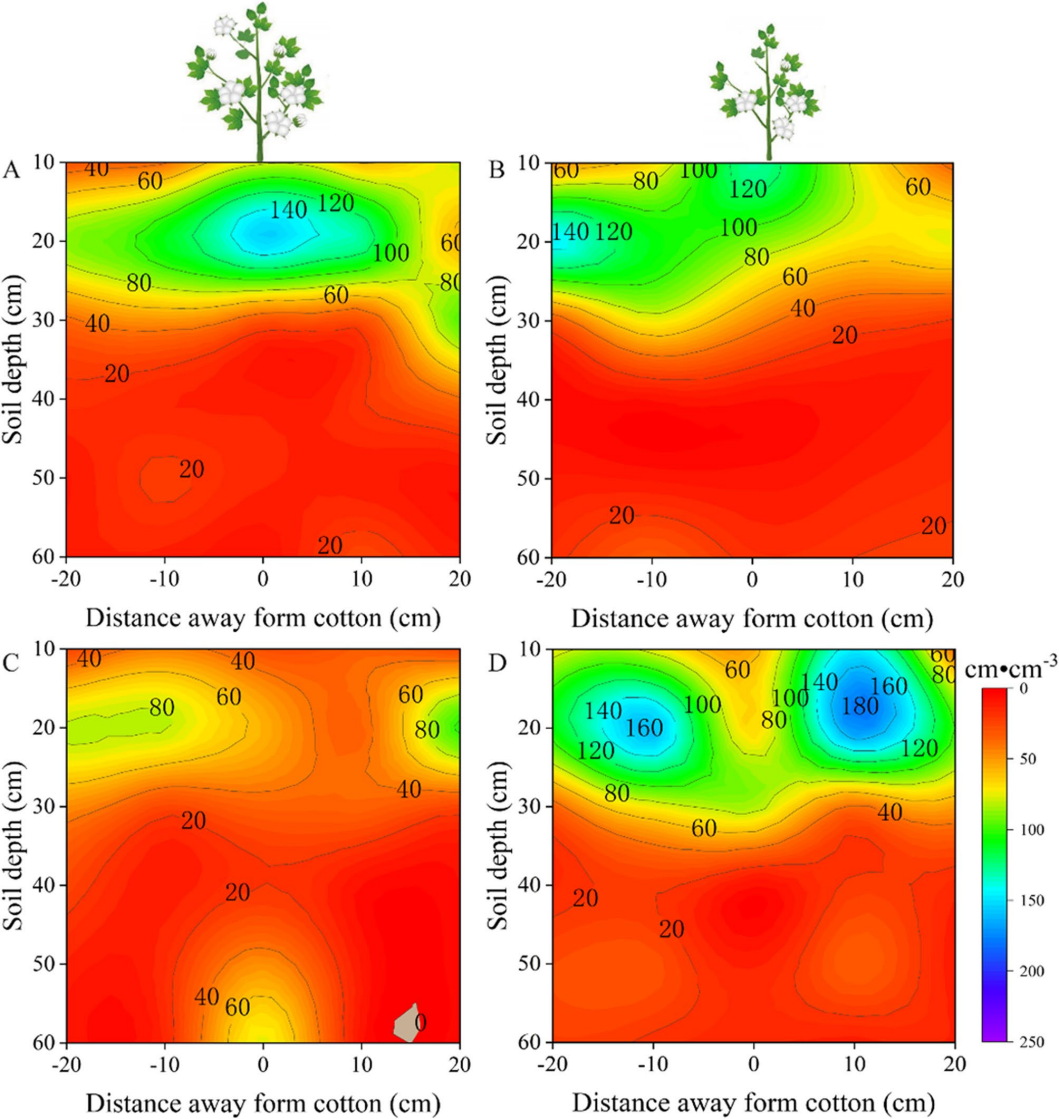
At the boll opening stage, vertical distribution of root biomass density in the soil profile was affected by soil water treatment. Root length density under control-drought sensitive cultivars (**A**), drought stress-drought sensitive cultivars (**B**), control-drought tolerant cultivars (**C**) and drought stress-drought tolerant cultivars (**D**) treatments, respectively. Values are the means ± SE (n = 6)

At the boll opening stage, there were differences in the root biomass density between the different treatments and among the different cultivars (Fig. 8). In addition, the biomass density under the control and drought treatments followed the same trend as that at the flower-boll stage, showing that the root biomass density was Mainly concentrated at 0–20 cm. Under control conditions, the root biomass density of the drought-sensitive variety was Mainly concentrated in the 0–30 cm soil layer (Fig. 8A), whereas the vertical root biomass density of drought-tolerant varieties was Mainly concentrated in the 0–20 cm layer of the soil (Fig. 8C). Under drought stress, the biomass density of drought-sensitive varieties was Mainly concentrated in the 0–20 cm layer of the soil (Fig. 8B), while the vertical root biomass density of drought-tolerant varieties was Mainly concentrated in the 0–30 cm layer of the soil (Fig. 8D). Compared to the control, the horizontal root biomass density in drought-tolerant cotton was wider than that under the drought stress treatment (Fig. 8BD). Compared with the control, the root biomass density of drought-sensitive varieties decreased by 5.16% under drought stress (Fig. 8AB), while the root biomass of drought-tolerant varieties increased significantly, by 70.75% (*p* < 0.05, Fig. 8CD).

## Discussion

A comprehensive assessment of crop drought tolerance necessitates consideration of various factors owing to the influence of intrinsic and extrinsic elements. Different crops have distinct drought-tolerant mechanisms, undermining reliance on a single indicator. Recent studies have detailed numerous traits associated with drought tolerance, spanning yield composition, plant height, tiller and spikelet numbers, grains per spike, thousand-grain weight, dry biomass, and other phenotypic traits [12–18]. Moreover, at the physiological and biochemical levels, parameters like photosynthetic intensity, chlorophyll content, abscisic acid accumulation, antioxidant enzyme activities, and osmotic regulatory substance accumulation stand as key indicators for evaluating drought tolerance [19–21]. However, published research utilizing root spatial distribution traits to evaluate crop drought 11tolerance have remained limited. Hence, the present study adopted a robust analytical approach, aiming to refine the precision and reliability of drought tolerance assessment by leveraging diverse indicators associated with both above-ground and root characteristics.

The *D*-value index integrates multiple root, physiological, and yield traits into a single quantitative measure of drought tolerance. Cultivars with high *D*-values generally exhibited root systems with greater vertical penetration, enabling plants to access deeper soil moisture reserves and maintain improved plant water status under drought conditions. These traits supported sustained photosynthetic activity, improved biomass accumulation, and reduced yield loss. In contrast, cultivars with low *D*-values tended to have roots concentrated in shallower soil layers, making them more vulnerable to surface soil desiccation and resulting in lower water uptake efficiency and greater yield reduction under drought. 

Under drought stress, plants employ various stress adaptation mechanisms, including root plasticity [22], i.e., adjusting root system architecture to acquire deep water resources [23]. In cotton, Luo et al. [24] found that mild and early-stage drought stress increased the length of lateral roots. In contrast, Lynch et al. [25] suggested that reducing the number of lateral roots under drought stress could mitigate metabolic costs to plants, thus optimizing the distribution of nutrient resources throughout the plant. Additionally, if a plant’s root system is too densely overlapped, this can affect the allocation of nutrient resources to favorable portions. This aligns with the findings of our present study, in which we observed that intermediate-sensitive cotton, when under drought stress, reduces its root growth to decrease metabolic costs and enhance drought tolerance. Furthermore, Bardhan et al. [26] revealed that when surface soil moisture is insufficient, abscisic acid inhibits the growth of primary roots of winter wheat, while plant hormones stimulate the growth of seeding roots into deeper soil layers. This corresponds to our present research, which revealed that drought-tolerant cotton encourages the growth of fine roots into deeper soil layers when faced with drought stress. In addition, we found that drought stress promoted the root-shoot ratio of cotton at anthesis and the boll stage. This is consistent with the vertical spatial stress of roots. VSSR promotes plant morphological adaptation mechanisms, such as significantly increasing the root-shoot ratio (*p* < 0.05).

The influence of drought stress on root system architecture primarily involves two traits: root angle and root deep. It has been previously suggested that a smaller root angle may be advantageous for crop survival in arid environments [27], as smaller root angles, fewer lateral roots, and deeper root systems can enhance drought tolerance in maize under drought stress [25]. In our present study, the results at the boll opening stage planting were consistent with these previous findings, indicating that drought stress can reduce root angles, with drought-tolerant cotton exhibiting smaller root angles under drought stress. However, these results differ from our findings at the flower-boll stage, in which we observed that drought-sensitive cultivars had a greater decrease in root angles compared to drought-tolerant cultivars. This discrepancy may be related to the rainfall occurring after July, which improved surface soil moisture. These findings emphasize the complexity of root system research and the need for in-depth exploration of the relationship between different root system types and their functions to gain a more comprehensive understanding. Focusing on research on individual root system traits and functions may provide partial information, but a comprehensive understanding of functional attributes requires further exploration of root system complexity.

To investigate the characteristics of root spatial distribution in response to drought stress in different cotton cultivars, we examined the root spatial distribution in the present field experiment at a depth of 0–60 cm. The depth selection was informed by prior research on the root spatial distribution in different crops. For example, Chen et al. [28] studied Maize root systems by excavating soil blocks to a depth of 60cm to analyze the relationship between fertilization depth, root systems, and yield. Additionally, Li et al. [29] investigated root distribution in intercropped and monocropped areas of wheat and Maize by excavating soil blocks to a depth of 100cm. These studies indicate that the Maize root spatial distribution Mainly occurs within the 0–60 cm soil layer, while wheat root systems extend up to 100 cm deep. Previous research has also delineated the primary range of cotton root spatial distributions. For example, Chen et al. [11] examined the cotton root distribution under drip irrigation within soil layers at a depth of 0–50 cm, noting the greatest variation in root density occurred at depths of 0–40 cm. Min et al. [30] excavated cotton roots down to a depth of 100 cm and found that root length density in the soil layers at depths of 0–60 cm accounted for approximately 83–95% of root tissue density. These prior findings serve as a foundation for our study, facilitating a deeper understanding of how the root systems of different cultivars respond to drought stress. In our present study, drought-tolerant cultivars exhibited Markedly greater root length density in deeper soil layers, showing an increase of 141.78% at the flower-boll stage and 147.96% at the boll-opening stage compared to drought-sensitive cultivars. These deep-root traits enhance access to stable subsoil moisture reserves, supporting leaf function and boll filling during stress recovery, and are consistent with the observed 24.25% yield advantage of drought-tolerant cultivars relative to drought-sensitive cultivars under drought stress.

The present study of cotton’s root spatial distribution has revealed some important findings, especially under conditions of limited soil moisture. Early studies have indicated that drought stress stimulates the growth of new roots deep in the soil, in particular, causing a significant increase in fine root density [6, 31]. This aligns with the results of our study, especially during the flower-boll stage. Under drought stress, drought-sensitive cultivars can increase root length density, while drought-tolerant cultivars exhibited higher root length density in the vertical distribution at depths of 30–60 cm. The increased root length density in deeper soil layers under drought conditions provides multiple physiological advantages. Firstly, deeper roots access relatively stable water reserves that are less affected by surface evaporation, thereby sustaining water uptake when upper soil layers dry out. This enhanced water acquisition strategy helps maintain higher leaf water potential, reduces stomatal closure, and thus sustains photosynthetic activity under drought stress. Secondly, improved plant water status supports cell turgor and metabolic functions, facilitating continued boll development and fiber elongation. Thirdly, deep-rooted agroecosystems exhibit reduced competition for nutrients in surface layers, thereby enabling more efficient nutrient uptake during reproductive stages. Collectively, these physiological benefits contribute to improved biomass accumulation and ultimately result in higher yield in drought-tolerant cultivars compared with drought-sensitive cultivars under water-limited conditions. Moreover, previous research also suggests that moderate drought stress can stimulate root growth in cotton, thus enhancing the drought tolerance of drought-tolerant cultivars and promoting crop growth recovery [32]. These trends are consistent with the results of our study, further emphasizing the correlation between root length density and crop growth.

Future research endeavors should therefore focus on unraveling the physiological mechanisms underpinning adaptive changes in the root spatial distribution and their consequential impact on plant growth and yield. Targeted genetic and molecular investigations could elucidate the advantages conferred by different cultivars or genotypes in their response to drought stress through adaptive plasticity in root structure at distinct growth stages. Subsequent studies could explore the link between root spatial distribution and root physiological characteristics. A more profound comprehension of the interplay between root morphology and functionality would enhance the current understanding of plant adaptive mechanisms to drought stress. The amalgamation of research spanning various tiers could furnish a more holistic understanding of plant root reactions to drought stress, providing a deeper genetic and physiological foundation for crop improvement. Additionally, prospective research utilizing advanced imaging and remote sensing techniques could evaluate plant root responses on a broader spatial spectrum. The fusion of ground-level observations with satellite-derived data might amplify our insight into the adaptive plasticity of plant roots to different geographical and environmental conditions, thereby furnishing more precise predictions and directives for future agricultural practices.

## Conclusion

In this study, our primary aim was to elucidate the impact of drought stress applied during the square stage on subsequent stages. Accordingly, we conducted principal component analysis, membership function analysis, and cluster analysis on data derived from different drought-tolerant cotton cultivars, classifying them into five categories. At the flower-boll stage, drought stress significantly reduced plant height, stem diameter, and SPAD and *F*_v_/*F*_m_ values, with a more pronounced effect on the root system. However, owing to the large amount of rainfall occurring in July 2021, the spatial distribution of roots did not show significant differences at the boll opening stage among cultivars. Specifically, at the flower-boll stage, drought conditions led to significant reduction in lateral root angles and the number of lateral roots for drought-tolerant, intermediate-sensitive, and drought-sensitive cultivars, while it also increased their root-shoot ratio. During this period, root length density of the 50–60 cm soil layer significantly increased in drought-tolerant cultivars compared to drought-sensitive ones, highlighting the drought-tolerant cultivars’ capacity to concentrate roots in deeper soil layers, which is crucial for cotton drought tolerance.

## Materials and methods

### Plant materials

This study focused on the evaluation of the responses of 30 cotton cultivars (Table 1), sourced from diverse geographical regions, to drought stress relative to well-watered conditions. Cultivars were selected to represent a broad geographical scope and a wide range of ecological adaptability, adequately reflecting their extensive distribution and genetic diversity. All cultivars were officially released commercial varieties obtained from national or provincial cotton breeding and extension institutes in China. Voucher information (variety name, authorized number, period, and breeding source) for each cultivar is provided in Table 1.

**Table 1:** Cultivars in cotton area from Yellow River Basin, China

No.	Variety	Authorizedunit	Authorizednumbers	Period	Sourceofcultivars
1	Jifeng 908	Jishenmian	2006	Period	Jimian 20 × 97G1
2	Jifeng 914	Guoshenmian	2,015,003	132d	Ji 668 × 97G1
3	YM111	Guoshenmian	20,170,002	124d	Han 6205×Han 802
4	Guoxinmian 9	Guoshenmian	2,009,004	120d	1275-23 × 1207
5	K836	Lushenmian	2,012,018	126d	Zhongmiansuo 12× K638
6	Lumian 522	Lushenmian	20,170,041	126d	K836 × 8891
7	Yuzaomian 9110	Yushenmian	2,012,006	121d	Yu 1335×Zhong 825
8	Cangmian 268	Lushenmian	20,160,030	106d	
9	Zhongmiansuo 41	Guoshenmian	2,002,001		Zhongmiansuo 2323 imported domestic Bt and CpTI
10	Xinshi 71,143	Guoshenmian	2,014,004	130d	B4-16 × 484
11	Zhongmiansuo 79	Yushenmian	2,010,006	123d	Zhongmiansuo 41×Zhong 4648
12	Cangmian 666	Lushenmian	20,160,030	123d	(Ji 589 × 612)×Ji 589
13	Shikang 126	Guoshenmian	2,008,002	119d	GK12×Shikang 389
14	Lumian 5172				
15	Jifeng 4	Guoshenmian	20,210,020		97–668 × 97G1
16	Jifeng 103	Jishenmian	20,190,008	131d	99 − 68 × 97G1
17	MH335223	Jishenmian	20,190,018	125d	Mei 8123×Ji 616
18	Jimian 262	Jishenmian	20,200,002	124d	Jihang 8×Feng 5328
19	Zhongmian 23	Guoshenmian	980,007	120d	(5658×Shan 5245)×4067×Zhongmiansuo 10×Jimian 6
20	Zhongmiansuo 50	Guoshenmian	2,007,013	132d	Bivalent Bt + CpTI gene was introduced into 394
21	Han 8266	Guoshenmian	2,014,001	110d	Han 4849×Han 5158
22	Nongda KZ05	Jishenmian	2,013,006	122d	Nongda 372×Nongda 20
23	Shandongxiamian11-42			123d	
24	Guoxinmian02			130-140d	
25	Xuzhou 1818	I3A01080		134d	Ningyou 18 × 088018
26	Guoxinmian05	Guoshenmian	2,006,003	132d	0106 × 82
27	Ji 228	Guoshenmian	2,008,003	125d	w112×Kangdan 258-1
28	K638	Lushenmian	2,012,018	124d	Shiyuan 321×Lumianyan 18
29	Chunbeibao			126d	
30	Dexiamian 1	Lushenmian	220		The excellent variants of Liao 3702 were selected and bred by systematic selection

### Experimental site description

**Fig. 9:**
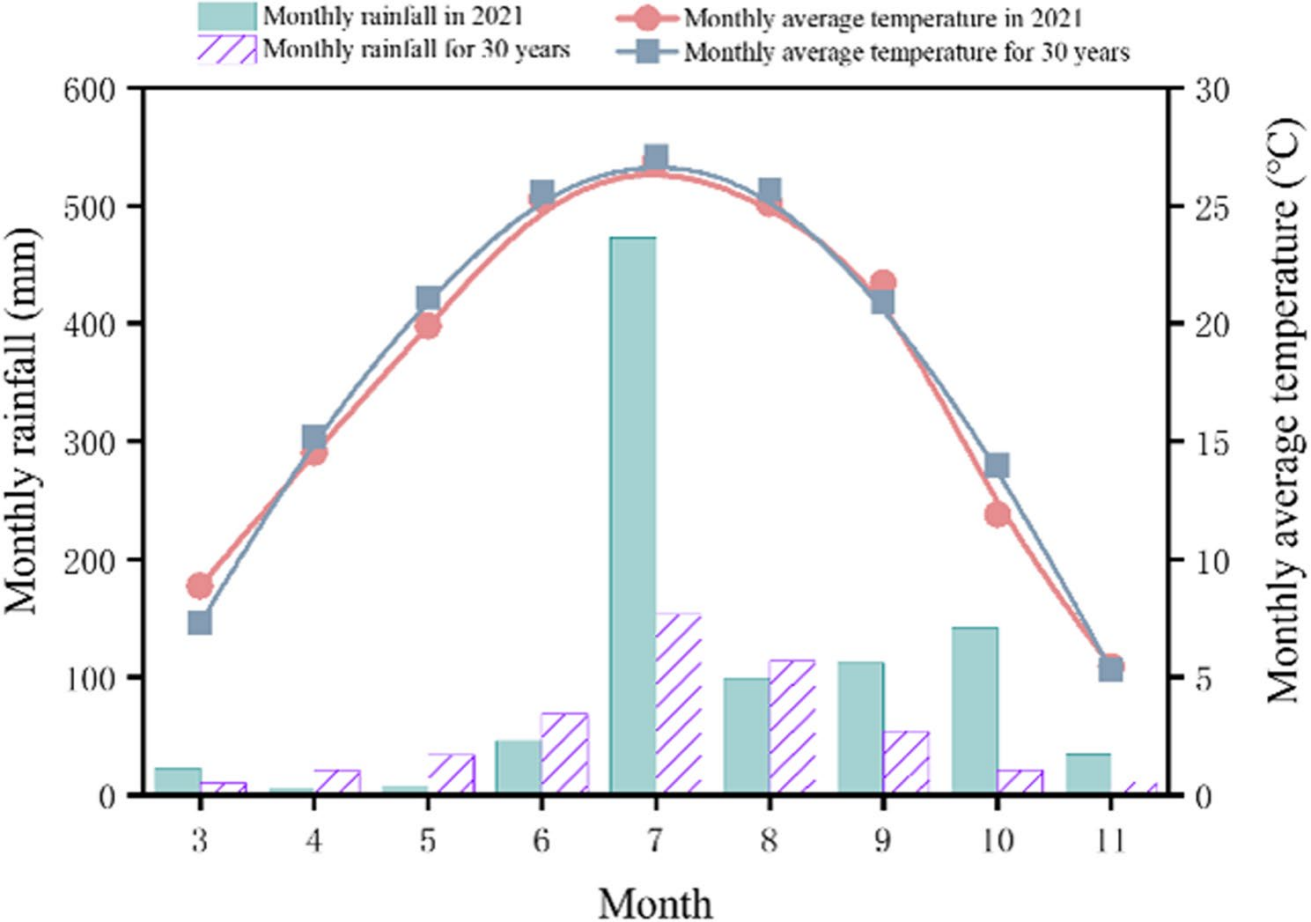
Rainfall and average temperature of Qingyuan Experimental Station in 2021 and 30yearsRainfall and average temperature of Qingyuan Experimental Station in 2021 and 30years

The experiment was conducted in May to October 2021 at the Qingyuan Experimental Station of the College of Agriculture, Hebei Agricultural University, Shiqiao Village, Qingyuan County, Baoding, Hebei, China (38.85°N, 115.30°E). The average annual temperature was 21.78 °C, and the annual Sunshine duration was 2091.4 h (Fig. 9). The soil type at the experimental site is loam with an organic Matter content of 15.72 g·kg^−1^, total nitrogen content of 1.10 g·kg^−1^, available phosphorus content of 15.11 mg·kg^−1^, available potassium content of 120.42 mg·kg^−1^, and alkali-hydrolyzable nitrogen content of 78.36 mg·kg^−1^. The soil relative water content of the study site is summarized in Fig. S1.

As cotton enters the budding stage, its growth accelerates, accompanied by a gradual rise in ambient temperature, resulting in increased water demand in the field. In the Yellow River Basin, drought stress is common during this period, leading to substantial evaporation of soil moisture. Consequently, ensuring timely irrigation during the budding stage (i.e., early June) becomes imperative for optimizing cotton yield. A two-factor split-plot design with three replicates (blocks) was used. An irrigation regime was established for main plots with two levels: control (CK, 1175 m^3^·hm^−2^, with 800 m^3^·hm^−2^ for base irrigation and 375 m^3^·hm^−2^ for square stage irrigation) and drought stress (DS, 822.5 m^3^·hm^−2^, with 560 m^3^·hm^−2^ for base irrigation and 262.5 m^3^·hm^−2^ for square stage irrigation). Border irrigation was utilized for both treatments. The irrigation volume was monitored using a water meter (with an accuracy of 0.0001 m^3^). Cultivars (30 genotypes) were assigned to subplots within each Main plot. In each block, Main-plot positions of CK and DS levels were randomized, and within every main plot, the 30 cultivars were arranged in a completely randomized arrangement; randomization was performed independently for each block.

To characterize plot-level soil water status, soil samples were collected from soil depths of 0–20, 20–40, 40–60, 60–80, and 80–100 cm before sowing and during the square, flowering, boll and boll-opening stages under both CK and DS levels. Gravimetric water content was determined by oven-drying, and the seasonal dynamics are shown in Fig. S1. For root-profile measurements, root-containing soil was sampled from depths of 0–60 cm at 10-cm increments (six layers) adjacent to target plants as described below.

Each subplot had an area of 10 m^2^ (row spacing, 76 cm; planting density, 75,000 plants·ha^−2^). Seeding was conducted Manually on April 25, 2021, with plastic film mulching applied. Thinning was conducted when the seedlings reached the two-leaf stage. Each plot received 450 kg·ha^−1^ of compound fertilizer containing 15% N, 15% P_2_O_5,_ and 15% K_2_O as base fertilizer. Additionally, 150 kg·ha^−1^ urea (46% N) was top dressed at flowering. Pest control, weed control, chemical control, and plant pruning were conducted in accordance with local agronomic guidelines.

**Fig. 10:**
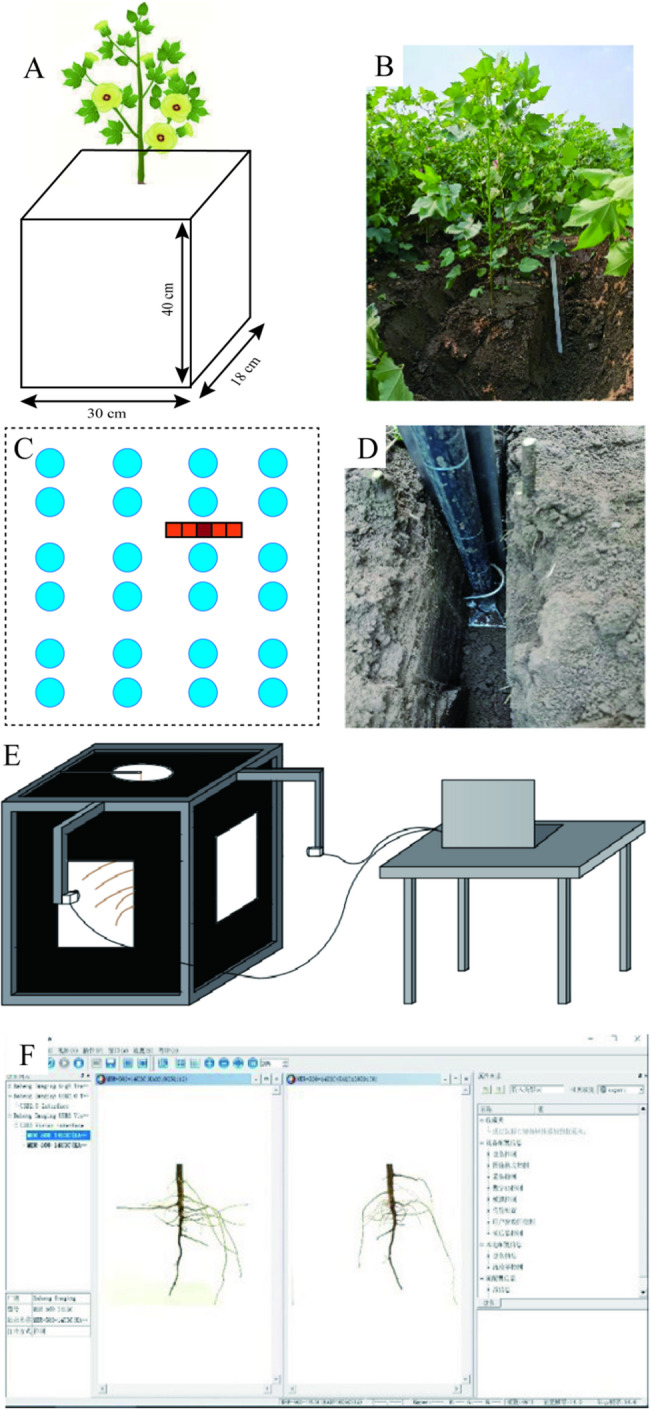
A holistic approach to root spatial distribution. Schematic diagram (**A**) and photograph (**B**) of root configuration sampling. Schematic diagram (**C**) and photograph (**D**) of root distribution at different soil depths. The root imaging system (**E**). Image processing with root estimator for Shovelomics traits (WinRHIZO) (**F**)

Sampling and measurementsPlant height and stem diameterAt flower-boll stage, five representative plants were selected from each plot for morphological assessments. Plant height was determined using a measuring tape. Stem diameter was gauged with a vernier caliper, specifically at a point 1 cm above the cotyledon node on the main stem. SPAD, *F*v/*F*m and Canopy temperatureAt flower-boll stage, five representative plants were selected from each plot. Leaf relative chlorophyll content (SPAD), maximum photosystem II quantum yield (*F*v/*F*m) and leaf temperature of the main stem functional leaf of the third were measured.At 9:00 ~ 11:00, the SPAD was determined using a SPAD-502 chlorophyll meter (Konica Minolta in Tokyo, Japan). Three readings were taken on each leaf to calculate the average SPAD value. In order to achieve a consistent and full dark adaptation of the leaf, *F*v/*F*m was measured from 10:00 pm to 12:00 pm. *F*v/*F*m was measured using an FMS-II chlorophyll fluorometer (Hansatech, UK), avoiding leaf veins and petioles. At 9:00 ~ 11:00, leaf temperature was measured using a hand-held infrared thermometer (AGRI-THERM II, Model 6110, USA). The sensor probe was positioned 5 cm away from the upper leaf, which was perpendicular to the direction of leaf unfolding. Temperature measurements were recorded as soon as the probe was stable [33]. Lateral root angle, number of lateral root and root/shoot ratioAt flower-boll and boll opening stage, three representative plants from each plot were chosen for root sampling using “Shovelomics” approach [34, 35]. The three-dimensional structure of the root system (Perpendicular to the sowing line direction and parallel to the sowing line direction) was analyzed [22]. Initially, pruning shears were used to sever the roots at the cotyledon node, and a soil block measuring 40 cm × 30 cm × 18 cm and centered around the cotton main stem was excavated with a shovel (Figs. 10A10B). The soil block containing the root system was extracted, lightly shaken to dislodge most of the adhering soil, and subsequently immersed in water with added detergent for 10 minutes to facilitate root-soil separation. A custom-made imaging device was then employed to capture images of the root system. Fig. 10C illustrates the root system image acquisition interface and the root system imaging device, while Fig. 10F showcases the software employed for obtaining images of the three-dimensional root architecture. The custom imaging device comprises a computer, two sets of industrial digital cameras (MER-500-14U3C; lens, M1224-MPW2), and an imaging darkroom. The computer must be equipped with a USB 3.0 interface, and the image sensors and lenses of the cameras were procured from Daheng (Group) Co., Ltd. These two industrial digital cameras (MER-500-14U3C) were mounted on a custom-made height-adjustable stand, strategically placed at two viewing windows within the imaging tent to ensure optimal imaging of the root system. The imaging software, Daheng Galaxy Viewer (x64), maintained consistent settings, including shooting position, exposure time, desired grayscale value, and white balance coefficient. Root system images were stored in BMP format in a designated folder. In a standard working environment, approximately 140 root system images were captured per hour, each image averaging about 14 mb in size. The acquired root system images were subsequently analyzed using RootNav software to extract data on root architecture, including number of lateral root and lateral root angle [36, 37]. Following the imaging of the root system, the underground portion was pruned at the cotyledon node, and both above-ground and below-ground parts were weighed for fresh weight, labeled, and placed in paper bags. Subsequently, they were blanched at 105°C for 30 minutes and dried in an 80°C oven for 48 hours to determine dry weight, which provided values for plant root/shoot ratios, shoot dry weight, and root dry weight indicators. Root length density and root biomass density At flower-boll and boll opening stage, three representative plants from each plot were measured for root spatial distribution of different depths (Figs. 10DE). A root box (10 cm×10 cm×7.5 cm) was used to collect root containing soil pieces in layers, with a maximum depth of 60 cm in the vertical direction and a total of six layers, namely 0-10 cm, 10-20 cm, 20-30 cm, 30-40 cm, 40-50 cm and 50-60 cm; the width of each layer was 50 cm in the horizontal direction and one layer was sampled every 10 cm (Fig. 10D).The soil pieces obtained were placed in sealed bags, numbered and stored in a refrigerator at 4°C for later analysis. Roots were rinsed by soaking the root-containing clods in surfactant-containing water for 1 h, stirring gently, then placing the clods in a sieve (0.2 mm^2^ aperture) and rinsing off the soil with a jet of water to remove the soil, and then picking out the contaminants to obtain the root samples from the clods. For root sample imaging, cleaned root samples were arranged within acrylic root trays containing distilled water, ensuring minimal overlap between roots. They were then subjected to scanning using an EPSON Expression 10000XL scanner. The scanned root images were subjected to analysis using WinRHIZO (Regent Instruments, Inc., Quebec City, Canada) root analysis software from Canada (Fig. 10F). This analysis facilitated the quantification of total root length and root volume, and so on [6, 38]. The roots were appropriately labeled, placed within paper bags, blanched at 105°C for 30 minutes, and subsequently dried within an 80°C oven for 48 h to ascertain their dry weight, thereby yielding the root dry weight. Ultimately, through meticulous calculation, the root length density (RLD, mg·cm^-3^) and root biomass density (RBD, cm·cm^-3^) were determined [39].The yield and yield components Cotton bolls were harvested from 20 plants in the center of each plot. The harvested cotton bolls were counted and weighed. Subsequently, the seed cotton from each harvest was collected, placed in nylon mesh bags, and stored in a dry room for 20 d for air drying. After the drying period, the seed cotton per plot was weighed to calculate the seed cotton yield.

### Statistical analysis

The following equations were used to evaluate the drought response of the 30 cotton cultivars.

1 $$\begin{aligned} &\:\text{D}\text{r}\text{o}\text{u}\text{g}\text{h}\text{t}\:\text{t}\text{o}\text{l}\text{e}\text{r}\text{a}\text{n}\text{c}\text{e}\:\text{c}\text{o}\text{e}\text{f}\text{f}\text{i}\text{c}\text{i}\text{e}\text{n}\text{t}\:\left(\text{\%}\right)=\\&\frac{\text{T}\text{h}\text{e}\:\text{a}\text{v}\text{e}\text{r}\text{a}\text{g}\text{e}\:\text{v}\text{a}\text{l}\text{u}\text{e}\:\text{o}\text{f}\:\text{t}\text{h}\text{e}\:\text{d}\text{r}\text{o}\text{u}\text{g}\text{h}\text{t}\:\text{s}\text{t}\text{r}\text{e}\text{s}\text{s}\:\text{i}\text{n}\text{d}\text{e}\text{x}}{\text{T}\text{h}\text{e}\:\text{a}\text{v}\text{e}\text{r}\text{a}\text{g}\text{e}\:\text{v}\text{a}\text{l}\text{u}\text{e}\:\text{o}\text{f}\:\text{t}\text{h}\text{e}\:\text{c}\text{o}\text{n}\text{t}\text{r}\text{o}\text{l}\:\text{i}\text{n}\text{d}\text{e}\text{x}} \end{aligned}$$

2 $$\:\mu\:\left({x}_{j}\right)=\frac{\left({x}_{j}-{x}_{min}\right)}{{(x}_{max}-{x}_{min})\:}\:\:\:\:\:\:\:\:j=\text{1,2},\cdots\:,n$$

3 $$\:{w}_{j}={p}_{j}{\sum\:}_{j=1}^{n}{p}_{j}\:\:\:\:\:\:\:\:\:\:\:\:\:\:\:\:\:j=\text{1,2},\cdots\:,n$$

4 $$\:D={\sum\:}_{j=1}^{n}\left[u\right({x}_{j})\times\:{w}_{j}]\:\:\:\:\:j=\text{1,2},\cdots\:,n$$

The drought tolerance coefficient ($\:{x}_{j}$) of each indicator and the value of the membership function (𝜇) of each indicator for each variety were calculated using Eq. (1) and Eq. (2), respectively. The drought tolerance coefficient reflects the relative performance of each cultivar under drought compared with control conditions, while the membership function standardizes different indicators to a comparable 0–1 scale, allowing integration across traits despite their different units and ranges. Based on the contribution of each composite indicator, the weights of the indicators were calculated using Eq. (3). The drought tolerance level, or *D*-value, of the different cotton cultivars was calculated using Eq. (4). A higher *D*-value indicates stronger integrated drought tolerance, characterized in this experiment by deeper root systems, greater water uptake capacity from deeper soil layers, high water-use efficiency, and better yield stability under drought stress. Additionally, 𝑥_𝑗_ is the *i*^th^ composite index; 𝑥_min_ is the minimum value of the *i*^th^ composite index; 𝑥_max_ is the maximum value of the *i*^th^ composite index; 𝑤_𝑗_ indicates the importance of the *i*^th^ composite index among all composite indices; 𝑝_𝑗_ is the contribution of the *i*^th^ composite index (CI) of each variety. The *D*-value is the comprehensive evaluation value representing the drought tolerance of each variety. Drought tolerance classes were assigned to the tested cotton cultivars based on their drought tolerance *D*-values [40, 41].

General data were recorded and sorted using Microsoft Excel 2019. SPSS 26.0 (IBM Corp., Armonk, NY, USA) was used for ANOVA. Plots were generated using Origin 2019b (OriginLab, Northampton, MA, USA) and GraphPad Prism 9.0 (GraphPad software, Inc., San Diego, CA, USA). Data are expressed as mean ± standard error (mean ± SE) One-way analysis of variance (ANOVA) and least significant difference tests were used to determine whether differences were significant at the 95% or 99% confidence level. 

## Supplementary Information

Supplementary Material 1.

## Data Availability

Data will be made available on request.
