# Supplementary material for: Optimizing root spatial distribution during the flower-boll stage can reduce yield losses from square stage drought in cotton
Source: BMC Plant Biol. 2025 Dec 15;25:1703. doi: 10.1186/s12870-025-07346-4 (PMC12706958; doi:10.1186/s12870-025-07346-4)
Supplement: Supplementary file 1 — Supplementary Material 1. [file 12870_2025_7346_MOESM1_ESM.docx]

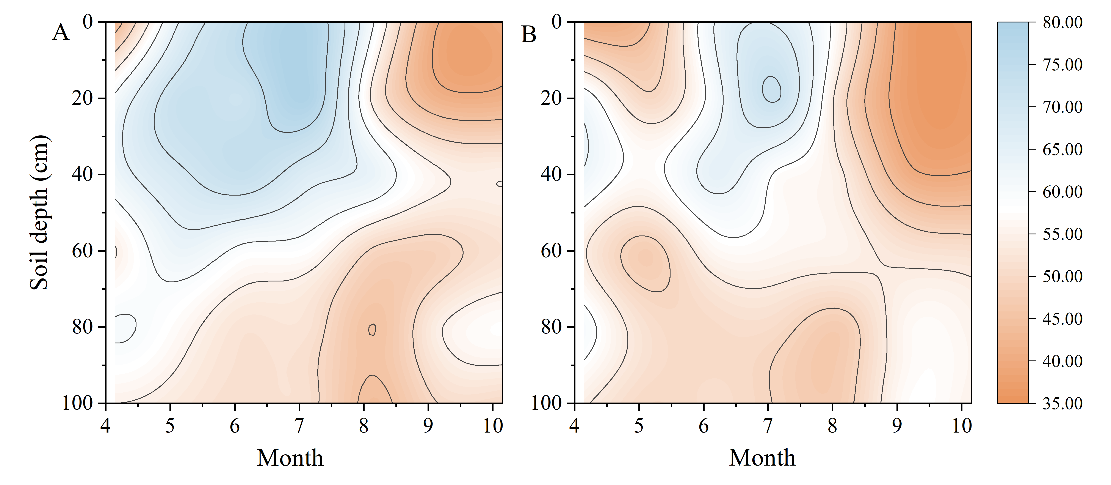


**Fig. S1** Soil relative water content in the cotton growing season (April to October) of Qingyuan Experimental Station in 2021. Control (A) and drought treatment (B), respectively.

**Table S1** Standardized values of drought tolerance coefficients for 30 cotton varieties

| Cultivar | PH | SD | SPAD | Fv/Fm | CT | R/S R | RGA | SDW | RDW | LRN | FLRA |
| --- | --- | --- | --- | --- | --- | --- | --- | --- | --- | --- | --- |
| Jifeng 908 | 1.01 | 1.11 | 1.06 | 1.00 | 0.98 | 0.75 | 0.77 | 1.10 | 0.82 | 1.30 | 1.15 |
| Jifeng 914 | 0.83 | 1.20 | 1.06 | 1.00 | 1.00 | 0.96 | 0.96 | 0.95 | 0.91 | 1.11 | 1.14 |
| YM111 | 0.75 | 0.92 | 1.05 | 1.00 | 0.97 | 0.48 | 0.49 | 1.47 | 0.81 | 1.09 | 1.23 |
| Guoxinmian 9 | 0.80 | 0.90 | 0.95 | 0.99 | 0.99 | 0.72 | 0.73 | 1.43 | 0.96 | 0.90 | 1.19 |
| K836 | 0.70 | 0.78 | 1.07 | 0.99 | 0.97 | 1.08 | 1.11 | 0.56 | 0.62 | 0.79 | 0.98 |
| Lumian 522 | 0.81 | 0.88 | 1.04 | 1.00 | 0.91 | 1.27 | 1.39 | 0.78 | 1.00 | 0.65 | 1.13 |
| Yuzaomian 9110 | 0.69 | 0.88 | 1.10 | 0.99 | 0.93 | 0.93 | 1.00 | 1.15 | 1.06 | 0.80 | 1.07 |
| Cangmian 268 | 0.86 | 0.87 | 1.04 | 1.00 | 0.94 | 1.01 | 1.07 | 0.92 | 0.93 | 0.64 | 1.08 |
| Zhongmiansuo 41 | 0.94 | 0.89 | 1.01 | 1.00 | 0.92 | 0.98 | 1.07 | 1.30 | 1.15 | 0.82 | 1.03 |
| Xinshi 71143 | 0.72 | 0.75 | 1.09 | 1.00 | 0.93 | 0.70 | 0.75 | 1.09 | 0.76 | 0.76 | 1.09 |
| Zhongmiansuo 79 | 0.82 | 0.94 | 1.09 | 1.00 | 0.94 | 1.06 | 1.13 | 0.88 | 0.95 | 0.81 | 0.94 |
| Cangmian 666 | 0.84 | 0.79 | 1.12 | 1.01 | 0.93 | 1.21 | 1.31 | 0.81 | 0.91 | 0.90 | 0.91 |
| Shikang 126 | 0.93 | 0.96 | 1.01 | 1.00 | 0.92 | 1.05 | 1.14 | 1.11 | 1.17 | 1.38 | 1.10 |
| Lumian 5172 | 0.73 | 0.85 | 1.10 | 1.00 | 0.93 | 1.07 | 1.15 | 0.73 | 0.76 | 1.23 | 0.98 |
| Jifeng 4 | 0.73 | 1.20 | 1.04 | 1.00 | 0.92 | 0.45 | 0.50 | 2.40 | 1.09 | 1.50 | 1.02 |
| Jifeng 103 | 0.76 | 0.80 | 1.06 | 0.98 | 0.90 | 0.85 | 0.95 | 0.56 | 0.47 | 0.71 | 0.90 |
| MH335223 | 0.77 | 0.86 | 1.03 | 0.98 | 0.89 | 0.82 | 0.92 | 0.97 | 0.81 | 0.86 | 0.93 |
| Jimian 262 | 0.89 | 1.29 | 1.00 | 0.99 | 0.94 | 1.02 | 1.09 | 0.97 | 1.00 | 1.22 | 0.99 |
| Zhongmian 23 | 0.89 | 0.81 | 1.12 | 0.98 | 0.99 | 0.72 | 0.73 | 0.92 | 0.66 | 0.94 | 0.95 |
| Zhongmiansuo 50 | 0.92 | 0.95 | 1.00 | 1.01 | 0.97 | 0.95 | 0.98 | 0.87 | 0.83 | 1.36 | 0.90 |
| Han 8266 | 0.86 | 0.98 | 0.95 | 0.96 | 0.97 | 0.82 | 0.85 | 0.97 | 0.83 | 1.23 | 0.97 |
| Nongda KZ05 | 0.73 | 0.93 | 1.03 | 0.96 | 0.96 | 0.91 | 0.95 | 0.76 | 0.68 | 0.77 | 0.91 |
| Shandongxiamian11-42 | 0.96 | 1.15 | 1.08 | 1.00 | 0.90 | 0.95 | 1.06 | 0.68 | 0.61 | 0.57 | 0.90 |
| Guoxinmian02 | 0.92 | 1.10 | 0.96 | 0.99 | 0.90 | 0.77 | 0.86 | 1.40 | 1.09 | 0.83 | 1.00 |
| Xuzhou 1818 | 1.08 | 0.96 | 0.99 | 1.00 | 1.03 | 0.76 | 0.74 | 1.10 | 0.73 | 1.13 | 0.91 |
| Guoxinmian05 | 0.92 | 0.89 | 0.99 | 0.99 | 0.99 | 0.76 | 0.76 | 0.98 | 0.82 | 1.36 | 0.97 |
| Ji 228 | 1.00 | 0.98 | 1.06 | 1.00 | 1.01 | 1.00 | 0.99 | 0.77 | 0.78 | 1.42 | 0.97 |
| K638 | 0.92 | 1.05 | 1.07 | 0.87 | 1.00 | 0.81 | 0.82 | 1.04 | 0.86 | 0.87 | 0.93 |
| Chunbeibao | 0.82 | 0.85 | 0.99 | 1.00 | 1.01 | 0.82 | 0.81 | 1.23 | 0.89 | 0.80 | 0.93 |
| Dexiamian 1 | 0.94 | 0.89 | 0.94 | 0.98 | 1.08 | 0.63 | 0.59 | 1.14 | 0.72 | 0.90 | 0.87 |

PH, Plant hight; SD, Stem diameter; LT, Leaf temperature; R/S R, Root/shoot ratio; LRA, Lateral root angle; SDW, Shoot dry weight; RDW, Root dry weight, LRN, Lateral roots number, FLRA, First order lateral root angle.

**Table S2** Load coefficients and cumulative contribution rate of each comprehensive index

| Index Index | Factor 1 | Factor 2 | Factor 3 | Factor 4 |
| --- | --- | --- | --- | --- |
| Factor weight | 0.41 | 0.26 | 0.20 | 0.13 |
| Eigenvalue | 3.28 | 2.09 | 1.67 | 1.06 |
| Coutributive ratio (%) | 29.82 | 19.04 | 15.15 | 9.61 |
| Cumulative contribution rate (%) | 25.27 | 43.94 | 62.45 | 73.63 |
| Eigenvector |  |  |  |  |
| Plant hight | 0.29 | -0.24 | 0.78 | 0.13 |
| Stem diameter | 0.45 | 0.28 | 0.49 | -0.29 |
| SPAD | -0.55 | 0.06 | -0.32 | 0.15 |
| Fv/Fm | -0.06 | 0.44 | -0.04 | 0.79 |
| Leaf temperature | 0.45 | -0.60 | 0.18 | 0.27 |
| Root-shoot ratio | -0.83 | 0.25 | 0.45 | 0.02 |
| Lateral root angle | -0.85 | 0.34 | 0.38 | -0.03 |
| Shoot dry weight | 0.80 | 0.41 | -0.25 | -0.14 |
| Root dry weight | 0.27 | 0.81 | 0.23 | -0.22 |
| Lateral roots number | 0.55 | 0.13 | 0.37 | 0.38 |
| First order lateral root angle | 0.25 | 0.63 | -0.28 | 0.17 |

**Table S3** Comprehensive index (CI) value

| Cultivar | CI1 | CI2 | CI3 | CI4 |
| --- | --- | --- | --- | --- |
| Jifeng 908 | -1.07 | 0.74 | 1.89 | 1.21 |
| Jifeng 914 | 0.06 | 1.01 | 0.82 | 0.94 |
| YM111 | -3.08 | 1.06 | -0.54 | 1.64 |
| Guoxinmian 9 | -1.97 | 1.45 | 0.34 | 0.60 |
| K836 | 1.56 | -2.09 | -2.35 | -0.16 |
| Lumian 522 | 2.90 | 0.88 | -2.04 | 0.79 |
| Yuzaomian 9110 | 0.53 | 1.07 | -2.32 | 0.68 |
| Cangmian 268 | 1.10 | 0.23 | -1.18 | 0.26 |
| Zhongmiansuo 41 | 0.73 | 1.65 | 0.08 | 0.45 |
| Xinshi 71143 | -0.69 | -0.47 | -2.61 | 0.95 |
| Zhongmiansuo 79 | 1.65 | -0.15 | -1.12 | 0.04 |
| Cangmian 666 | 2.82 | -0.77 | -1.82 | 0.61 |
| Shikang 126 | 0.89 | 2.12 | 1.08 | 1.50 |
| Lumian 5172 | 1.64 | -0.80 | -1.70 | 1.00 |
| Jifeng 4 | -3.82 | 3.80 | 1.21 | 1.26 |
| Jifeng 103 | 0.98 | -2.54 | -2.42 | -1.29 |
| MH335223 | 0.35 | -0.25 | -1.51 | -0.60 |
| Jimian 262 | 0.79 | 1.65 | 1.84 | -0.23 |
| Zhongmian 23 | -0.65 | -1.99 | -0.49 | -0.25 |
| Zhongmiansuo 50 | 0.34 | -0.55 | 1.59 | 0.44 |
| Han 8266 | -0.81 | -0.03 | 1.48 | -1.22 |
| Nongda KZ05 | 0.46 | -1.42 | -1.19 | -1.74 |
| Shandongxiamian11-42 | 1.71 | -1.09 | -0.45 | -1.03 |
| Guoxinmian02 | -0.67 | 2.17 | 1.05 | -0.65 |
| Xuzhou 1818 | -1.21 | -1.23 | 2.97 | -0.04 |
| Guoxinmian05 | -1.11 | -0.57 | 1.72 | 0.32 |
| Ji 228 | 0.52 | -0.96 | 2.04 | 0.69 |
| K638 | -0.72 | -0.68 | 0.94 | -4.67 |
| Chunbeibao | -0.85 | -0.44 | 0.19 | -0.08 |
| Dexiamian 1 | -2.39 | -1.82 | 2.52 | -1.41 |

**Table S4** The value of each cultivars μ (X_j_)

| Cultivar | U（X1） | U（X2） | U（X3） | U（X4） | D-value | Rank |
| --- | --- | --- | --- | --- | --- | --- |
| Jifeng 908 | 0.41 | 0.52 | 0.81 | 0.93 | 0.59 | 10 |
| Jifeng 914 | 0.58 | 0.56 | 0.62 | 0.89 | 0.62 | 5 |
| YM111 | 0.11 | 0.57 | 0.37 | 1.00 | 0.40 | 25 |
| Guoxinmian 9 | 0.28 | 0.63 | 0.53 | 0.84 | 0.49 | 20 |
| K836 | 0.80 | 0.07 | 0.05 | 0.71 | 0.45 | 23 |
| Lumian 522 | 1.00 | 0.54 | 0.10 | 0.86 | 0.68 | 3 |
| Yuzaomian 9110 | 0.65 | 0.57 | 0.05 | 0.85 | 0.53 | 15 |
| Cangmian 268 | 0.73 | 0.44 | 0.26 | 0.78 | 0.56 | 12 |
| Zhongmiansuo 41 | 0.68 | 0.66 | 0.48 | 0.81 | 0.65 | 4 |
| Xinshi 71143 | 0.47 | 0.33 | 0.00 | 0.89 | 0.39 | 27 |
| Zhongmiansuo 79 | 0.81 | 0.38 | 0.27 | 0.75 | 0.58 | 11 |
| Cangmian 666 | 0.99 | 0.28 | 0.14 | 0.84 | 0.61 | 6 |
| Shikang 126 | 0.70 | 0.74 | 0.66 | 0.98 | 0.74 | 1 |
| Lumian 5172 | 0.81 | 0.27 | 0.16 | 0.90 | 0.55 | 13 |
| Jifeng 4 | 0.00 | 1.00 | 0.69 | 0.94 | 0.52 | 16 |
| Jifeng 103 | 0.71 | 0.00 | 0.03 | 0.54 | 0.37 | 30 |
| MH335223 | 0.62 | 0.36 | 0.20 | 0.65 | 0.47 | 21 |
| Jimian 262 | 0.69 | 0.66 | 0.80 | 0.70 | 0.70 | 2 |
| Zhongmian 23 | 0.47 | 0.09 | 0.38 | 0.70 | 0.38 | 28 |
| Zhongmiansuo 50 | 0.62 | 0.31 | 0.75 | 0.81 | 0.59 | 9 |
| Han 8266 | 0.45 | 0.40 | 0.73 | 0.55 | 0.51 | 19 |
| Nongda KZ05 | 0.64 | 0.18 | 0.25 | 0.46 | 0.42 | 24 |
| Shandongxiamian11-42 | 0.82 | 0.23 | 0.39 | 0.58 | 0.55 | 14 |
| Guoxinmian02 | 0.47 | 0.74 | 0.66 | 0.64 | 0.60 | 8 |
| Xuzhou 1818 | 0.39 | 0.21 | 1.00 | 0.73 | 0.51 | 17 |
| Guoxinmian05 | 0.40 | 0.31 | 0.78 | 0.79 | 0.51 | 18 |
| Ji 228 | 0.65 | 0.25 | 0.83 | 0.85 | 0.61 | 7 |
| K638 | 0.46 | 0.29 | 0.64 | 0.00 | 0.39 | 26 |
| Chunbeibao | 0.44 | 0.33 | 0.50 | 0.73 | 0.46 | 22 |
| Dexiamian 1 | 0.21 | 0.11 | 0.92 | 0.52 | 0.37 | 29 |
